# Guidance for Triangulating Data and Estimates of HIV Prevalence Among Pregnant Women and Coverage of PMTCT Using the Spectrum AIDS Impact Module

**DOI:** 10.1097/QAI.0000000000003514

**Published:** 2024-11-05

**Authors:** Magdalene K. Walters, Eline L. Korenromp, Anna Yakusik, Ian Wanyeki, André Kaboré, Arthur Poimouribou, Célestine Ki, Coumbo Dao, Paul Bambara, Salam Derme, Théophile Ouedraogo, Kai Hon Tang, Marie-Claude Boily, Mary Mahy, Jeffrey W. Imai-Eaton

**Affiliations:** a MRC Centre for Global Infectious Disease Analysis, School of Public Health, Imperial College London, London, United Kingdom;; b Data for Impact Department, Joint United Nations Programme on HIV/AIDS, Geneva, Switzerland;; c Data for Impact Division, Joint United Nations Programme on HIV/AIDS, Ouagadougou, Burkina Faso;; d Permanent Secretary of the National Council for the Fight Against AIDS and Communicable Infections, Burkina Faso;; e Direction de la Santé de la Famille (DSF), Ouagadougou, Burkina Faso; and; f Center for Communicable Disease Dynamics, Department of Epidemiology, Harvard T.H. Chan School of Public Health, Boston, MA.

**Keywords:** HIV, AIDS impact module, pregnant women, ART, PMTCT

## Abstract

**Background:**

Most countries use the Spectrum AIDS Impact Module (Spectrum-AIM), antenatal care routine HIV testing, and antiretroviral treatment data to estimate HIV prevalence among pregnant women. Nonrepresentative program data may lead to inaccurate estimates of HIV prevalence and treatment coverage for pregnant women.

**Setting:**

One hundred fifty-four countries and subnational locations across 126 countries.

**Methods:**

Using 2023 UNAIDS HIV estimates, we calculated 3 ratios: (1) HIV prevalence among pregnant women to all women 15–49 yrs (prevalence), (2) ART coverage before pregnancy to women 15–49 yrs ART coverage (ART prepregnancy), and (3) ART coverage at delivery to women 15–49 yrs ART coverage (PMTCT coverage). We developed an algorithm to identify and adjust inconsistent results within regional ranges in Spectrum-AIM, illustrated using Burkina Faso estimates.

**Results:**

In 2022, the mean regional ratio of prevalence among pregnant women to all women ranged from 0.68 to 0.95. ART coverage prepregnancy ranged by region from 0.40 to 1.22 times ART coverage among all women. Mean regional PMTCT coverage ratios ranged from 0.85 to 1.51. The prevalence ratio in Burkina Faso was 1.59, above the typical range 0.62–1.04 in western and central Africa. Antenatal clinics reported more PMTCT recipients than estimated HIV-positive pregnant women from 2015 to 2019. We adjusted inputted PMTCT program data to enable consistency of HIV prevalence among pregnant women from programmatic routine HIV testing at antenatal clinics with values typical for western and central Africa.

**Conclusions:**

These ratios offer Spectrum-AIM users a tool to gauge the consistency of their HIV prevalence and treatment coverage estimates among pregnant women with other countries in the region.

## Abstract 2

Supplemental Digital Content is Available in the Text.

## INTRODUCTION

Estimates of HIV prevalence among pregnant women determine need for and coverage of antiretrovirals for prevention of maternal to child transmission (PMTCT), a key input to estimating pediatric HIV infections and monitoring progress toward eliminating mother-to-child HIV transmission.^1–4^ Most countries estimate the number of pregnant women living with HIV (PWLHIV) and PMTCT coverage using the AIDS Impact Module in Spectrum (Spectrum-AIM). Comparing related model outcomes and typical patterns across locations can reveal inconsistencies in input data or model assumptions that may result in inaccurate estimates.

Country-specific estimates of HIV prevalence among pregnant women are the result of a multi-step modeling process in Spectrum-AIM. The number of PWLHIV is calculated by multiplying the age-specific number of women living with HIV (WLHIV) in reproductive ages (15–49 years [15–49 yrs]), age-specific fertility rates (by 5-year age group), and the relative fertility of WLHIV relative to women without HIV (fertility rate ratio [FRR]). Age-specific HIV prevalence is derived by fitting a mathematical model to local HIV surveillance data,^5–7^ including HIV prevalence from household surveys, prevalence among pregnant women attending antenatal clinics (ANC), prevalence surveys among key populations, new HIV diagnoses, or AIDS-related deaths. Age-specific fertility rates for each country are obtained from the United Nations World Population Prospects.^8^ The Spectrum-AIM model reflects that fertility rates among WLHIV are different than among women without HIV. Relative fertility of WLHIV compared with women without HIV by age, treatment status, and local effects are estimated from national household surveys in African countries that measure HIV status and collect birth histories.^8,9^ These FRRs are applied to all countries, including those outside of Africa. The number of women who acquire HIV infection during pregnancy or breastfeeding are calculated by applying age-specific incidence rates to the number of HIV-negative pregnant or breastfeeding women. Women who acquire HIV during pregnancy and breastfeeding are exposed to a higher vertical transmission probability, reflecting the period of high HIV viraemia after seroconversion.^10^ Inconsistencies in population prevalence, fertility rates, or relative fertility among WLHIV may yield inaccurate estimates of HIV prevalence and treatment coverage among pregnant women within Spectrum-AIM.

Quality of information about HIV among pregnant women varies by region and epidemic type.^11–13^ In most African countries with high HIV prevalence, ANC attendance and routine HIV testing at ANC (ANC-RT) before delivery is nearly universal. Routinely reported data on the HIV status of women at entry to ANC for a given pregnancy provide direct measures of HIV prevalence among pregnant women. Spectrum-AIM calculates a “local adjustment factor” (LAF) to calibrate the modeled prevalence among pregnant women to HIV prevalence measured from national ANC-RT. The default LAF value of 1.0 indicates that the fertility of WLHIV is consistent with the default HIV FRR estimated from household survey data. In the 2023 UNAIDS estimates, most sub-Saharan African (SSA) locations (n = 36/43) calibrated the LAF to ANC-RT data.^14,15^ The default FRRs derived from household surveys in SSA countries may mis-specify fertility of WLHIV in other regions, which have smaller HIV epidemics and new infections more concentrated among selected populations who may have different fertility.^16–19^ In absence of representative measures of HIV prevalence among pregnant women, some Spectrum-AIM users manually adjust the LAF to ensure modeled estimates of PWLHIV are greater than numbers of PWLHIV receiving ARVs from program data. In non-SSA regions, in 2023 UNAIDS HIV estimates, 30 countries adopted the SSA-based default FRR, 24 countries fit the LAF to HIV testing from ANC-RT, and 29 countries manually set the LAF so that it produced PMTCT coverage consistent with the overall epidemic (ie, ensuring the estimated number of PWLHIV exceeded the program-reported number of PWLHIV receiving ART).^14,15^

Large adjustments to the default fertility patterns or uncharacteristically large differences between related model outputs could indicate inaccurate model estimates, inaccurate input data, or inappropriate interpretations of data sources. To identify possible discrepancies between model assumptions and data, we used UNAIDS estimates from Spectrum-AIM published in 2023 to establish patterns for HIV prevalence and treatment coverage among pregnant women for each region. We also proposed an algorithm to guide Spectrum-AIM users to compare and align model assumptions with regional patterns, surveillance data, and programmatic input.^14,15^ We applied the algorithm to program data and Spectrum-AIM estimates reported by Burkina Faso. Burkina Faso was chosen as a case study due to atypically high reported HIV prevalence among ANC clients relative to total population prevalence and reporting more women receiving ART for PMTCT than total HIV-positive pregnant women from the model (PMTCT coverage over 100%).

## METHODS

We used Spectrum-AIM files submitted by HIV estimation teams to UNAIDS for publication in 2023 to calculate 3 ratios: (1) HIV prevalence among pregnant women to HIV prevalence among all women 15–49 yrs (“prevalence ratio”), (2) ART coverage among PWLHIV before the current pregnancy to ART coverage among WLHIV 15–49 yrs (“ART coverage prepregnancy ratio”), and (3) the ratio of ART coverage in PWLHIV at delivery to ART coverage in WLHIV 15–49 yrs (“PMTCT coverage ratio”). We reported these ratios for the year 2022, as they did not vary substantially over the past decade. We summarized ranges for each ratio by UNAIDS region. Values outside of these ranges may indicate inconsistent data for the number of pregnant women on ART, number of PWLHIV, or low predictive power of the local HIV surveillance data to estimate HIV prevalence in the general population.

### Data Sources

We extracted outputs from 154 publicly available national or subnational publicly available Spectrum-AIM HIV estimates files submitted by 126 countries and published by UNAIDS in 2023.^15^ Three SSA countries had subnational files (Kenya, Zimbabwe, Ethiopia); the remaining 123 files represented national HIV epidemics created by country teams and submitted to UNAIDS for review and publication. Spectrum-AIM methods are described elsewhere.^20–22^ Briefly, in countries with high HIV prevalence (n= 64, mostly in SSA, including Burkina Faso), adult HIV prevalence and incidence trends were estimated from data on HIV prevalence among (1) nationally representative samples of adults from household-based surveys and (2) pregnant women attending ANC, sampled in periodic sentinel surveillance up to the mid-2010s and more recently from ANC-RT data.^5^ In countries with lower HIV prevalence, epidemic trends were fit to national HIV and/or AIDS case reports and AIDS-related deaths reported through vital registration with the CSAVR or ECDC models (n = 46) or from HIV prevalence survey and surveillance data among risk groups using the EPP concentrated (n = 27) or AEM models (n = 12).^6,7^ All incidence models accounted for the effects of ART on survival and transmission. Locations and the estimation method for each are reported in Table S1, Supplemental Digital Content, http://links.lww.com/QAI/C344.

Spectrum-AIM calculates HIV prevalence among pregnant women from age-specific HIV prevalence of all women 15–49 yrs, age-specific fertility rates,^8^ and HIV fertility rate ratios.^9^ Data on HIV FRRs outside SSA are limited; therefore, default HIV FRR patterns for SSA are applied to all regions, despite very different risk populations and contraceptive use. We extracted Spectrum-AIM's estimates for HIV prevalence among women 15–49 yrs, HIV prevalence among pregnant women, ART coverage among WLHIV 15–49 yrs, and initiation timing for PWLHIV on ART for 2022. We compared HIV prevalence among pregnant women and women 15–49 yrs to nationally representative surveys where available (see Table S2 and Fig. S1, Supplemental Digital Content, http://links.lww.com/QAI/C344).

### HIV Prevalence, ART, and PMTCT Ratios

We calculated the “prevalence ratio” for all Spectrum-AIM files by dividing the HIV prevalence among pregnant women by the HIV prevalence among women 15–49 yrs (Equation 1). Values larger than 1 indicate that the HIV prevalence among pregnant women is higher than HIV prevalence among women aged 15–49 yrs.

$$\text{Prevalence}\;\text{ratio}=\frac{\text{HIV}\;\text{prevalence}\;\text{among}\;\text{pregnant}\;\text{women}}{\text{HIV}\;\text{prevalence}\;\text{among}\;\text{women}\;15-49\;\text{yrs}}$$ (1)

The “ART coverage prepregnancy ratio” was calculated as the proportion of PWLHIV on ART before the current pregnancy from programmatic input and Spectrum-AIM's estimate of PWLHIV, divided by ART coverage among WLHIV 15–49 yrs (Equation 2). Values larger than 1 indicate that ART coverage before pregnancy is higher than ART coverage among WLHIV 15–49 yrs.

$$\text{ART}\;\text{coverage}\;\text{pre}-\text{pregnancy}\;\text{ratio}=\frac{\text{ART}\;\text{coverage}\;\text{before}\;\text{pregnancy}\;\text{among}\;\text{all}\;\text{PWLHIV}}{\text{ART}\;\text{coverage}\;\text{for}\;\text{all}\;\text{WLHIV}\;\text{aged}\;15-49\;\text{yrs}}$$ (2)

The “PMTCT coverage ratio” was the ratio of total ART coverage (ie, proportion of PWLHIV who received ART during the pregnancy, started before or during the current pregnancy) and ART coverage for WLHIV 15–49 yrs (Equation 3).^23^ Values larger than 1 indicate that ART coverage at delivery is higher than ART coverage among WLHIV 15–49 yrs.

$$\text{PMTCT}\;\text{coverage}\;\text{ratio}=\frac{\text{ART}\;\text{coverage}\;\text{at}\;\text{delivery}\;\text{amongst}\;\text{all}\;\text{PWLHIV}}{\text{ART}\;\text{coverage}\;\text{for}\;\text{all}\;\text{WLHIV}\;\text{aged}\;15-49\;\text{yrs}}$$ (3)

### Identifying Typical Ranges and Outliers by Region

We calculated the mean of each ratio by UNAIDS-defined regions, broadly representing variations in epidemic type: eastern and southern Africa (ESA), western and central Africa (WCA), Latin America and Caribbean (LAC), Asia and Pacific (AP), eastern Europe and central Asia (EECA), western and central Europe and North America (WCENA), middle East and northern Africa (MENA). For ESA and WCA, we considered location-specific ratios between 0.75 times lower to 1.25 times higher than the regional mean as “typical” and those outside this range to be outliers. For other regions, we present these ranges but emphasize the influence of local HIV surveillance data on estimating HIV prevalence among pregnant women. Alternate methods to define typical ranges were considered (see S1 and Table S4, Supplemental Digital Content, http://links.lww.com/QAI/C344).

We calculated a typical LAF by region as the average LAF weighted according to how close location-specific ratios (Ratio_L_) were to the regional mean ratio (Ratio_R_). The inverse favors values that are closest to the regional mean ratio. Weighted regional LAFs were calculated separately for all ratios (see Table S5, Supplemental Digital Content, http://links.lww.com/QAI/C344).

$$w_{L}=\frac{1}{\left|{\text{Ratio}}_{L}-{\text{Ratio}}_{R}\right|}$$ (4)

$${\text{LAF}}_{R}=\frac{\sum_{C}w_{L}\times {\text{LAF}}_{L}}{\sum_{C}w_{L}}$$ (5)

In addition, we identified inconsistent ART coverage among WLHIV 15–49 yrs and PWLHIV. Inconsistent coverage estimates consisted of (1) reporting more pregnant women receiving ART than estimated pregnant WLHIV and (2) large year-to-year differences in the number of pregnant women receiving ART. The first indicated inaccurate program data or an inaccurate estimate of pregnant WLHIV depending on the location. The second indicated possible inaccurate reporting of program data or atypical programmatic changes that should be verified by those familiar with the program implementation.

### Implications of Typical Patterns and Outliers by Region

For countries where transmission primarily occurs outside of key populations, we expected prevalence ratios to be less than 1 (ie, lower HIV prevalence among pregnant women than all women 15–49 yrs),^24,25^ and the ART prepregnancy ratios (on ART before pregnancy) to be less than 1, because PWLHIV are on average younger and so acquired HIV more recently than nonpregnant WLHIV.^26,27^ We expected PMTCT coverage ratios to be above 1 in countries with high ANC-RT coverage and ART for PMTCT uptake, as pregnant women who were untreated before pregnancy should be diagnosed and initiated on ART through ANC-RT.^28^

Outside of SSA, where HIV transmission is mostly among key populations and their partners, and fertility and contracepting patterns are different from SSA, there was little *a priori* information about expected typical relationships for the prevalence, ART prepregnancy, or PMTCT ratios. In non-SSA countries that fit the LAF to HIV prevalence reported through ANC-RT, there tend to be fewer years reporting HIV prevalence among pregnant women. Thus, estimates of HIV prevalence among pregnant women from these countries are likely to be less certain and more varied than SSA countries using ANC-RT data to fit the LAF.

Across all regions, we further hypothesized that countries with outlier prevalence, ART coverage, or PMTCT coverage ratios had atypical LAFs that were fitted or modified to reconcile discrepant data on HIV prevalence among pregnant women (whether fit to ANC-RT data or estimated by Spectrum-AIM) and the number of PWLHIV on ART from programs (see Fig. S2 and S3, Supplemental Digital Content, http://links.lww.com/QAI/C344). Alternatively, if the distribution of LAFs across countries within a region systematically differed from 1, this may indicate that the regional HIV-related FRR parameters in Spectrum-AIM are mis-specified.

### Guidance for Reviewing Data and Model Outputs With Burkina Faso Case Study

**FIGURE 1.**
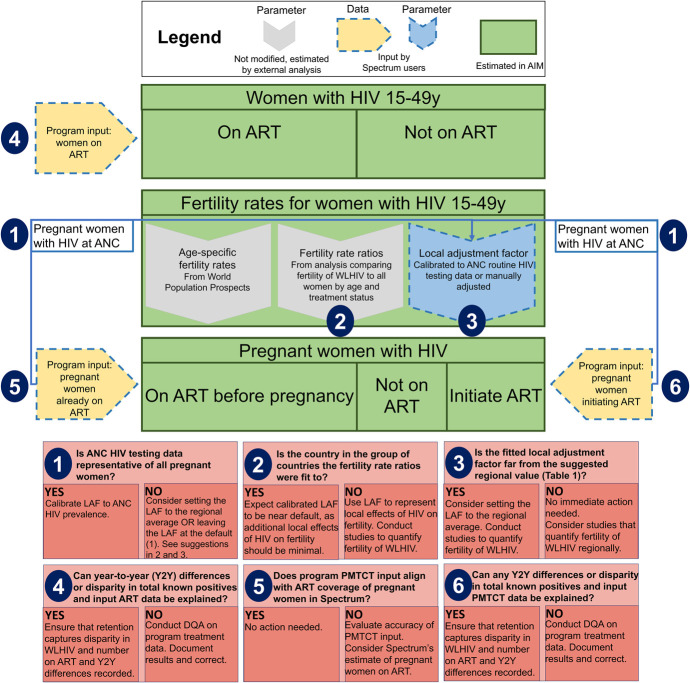
Recommendations to align Spectrum-AIM estimates of HIV prevalence and treatment coverage of pregnant women with regional means.

From the outlier analysis, we developed a 6-step algorithm to assess data and model assumptions regarding HIV prevalence and ART coverage among pregnant women (Fig. 1). We applied the algorithm using a preliminary Spectrum-AIM file produced by Burkina Faso (WCA region) for the 2023 round of UNAIDS published estimates. This file represented the most up-to-date surveillance and treatment data from the national HIV program and estimates available as of 2023.

This study was reviewed and approved by the Research Governance and Integrity team of Imperial College London (ICREC #6300528).

## RESULTS

### Prevalence, ART Prepregnancy, and PMTCT Ratios

**FIGURE 2.**
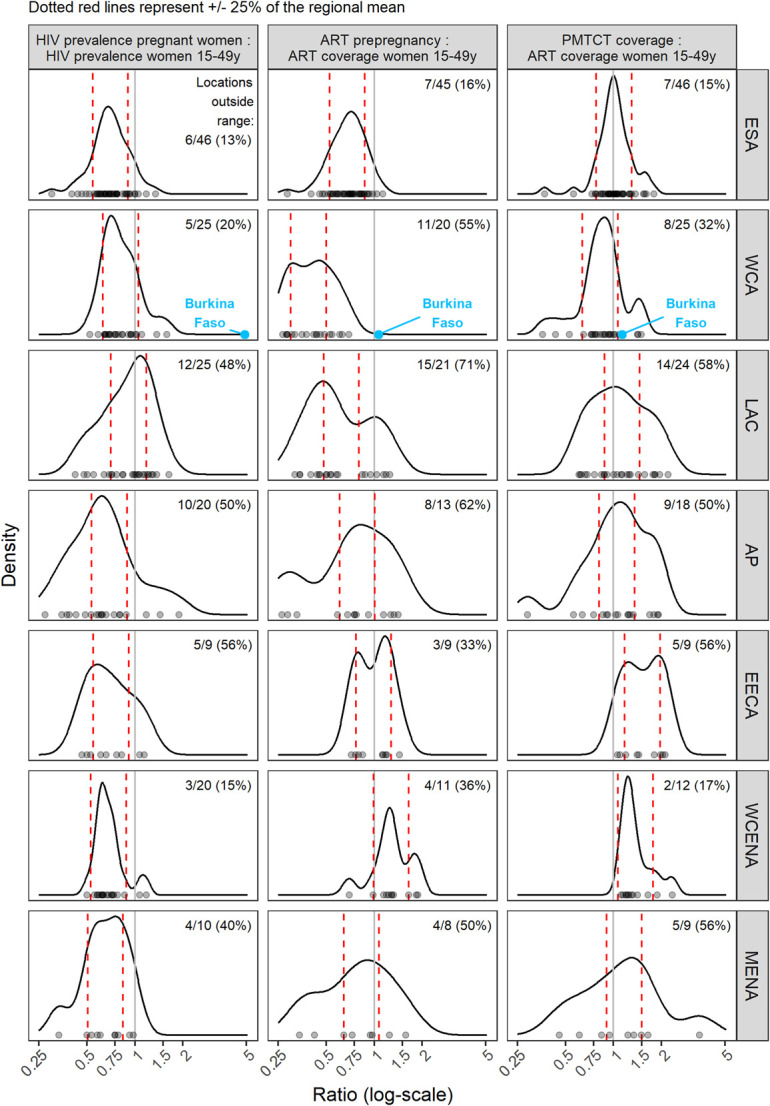
Prevalence, ART prepregnancy, and PMTCT ratio by region. Points on the x-axis represent distribution of ratios for all locations. Red dashed lines represent typical ranges of each ratio by region calculated as 0.75–1.25 times the mean value. Curved lines show density of ratios by region, with more common ratios represented as peaks. Multimodal distributions represent within region heterogeneity of ratios, meaning the “typical” ranges should be interpreted with caution.

**FIGURE 3.**
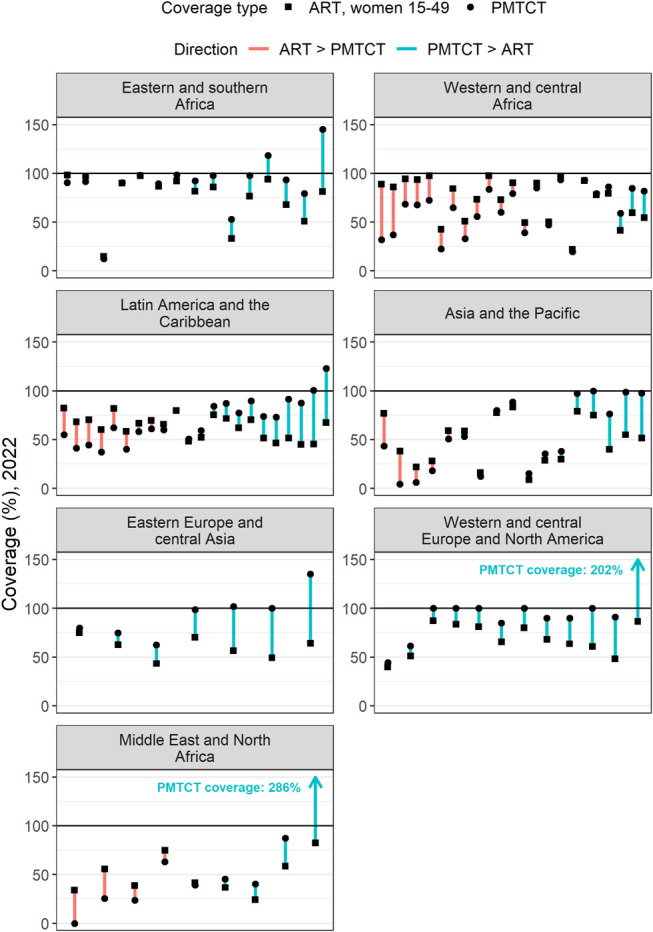
Difference between ART coverage among WLHIV 15–49 yrs and ART coverage at delivery in 2022 for all countries analyzed (grouped by region). Length of line indicates size of difference for a given country, color of line indicates whether ART coverage or PMTCT coverage was higher, and shape of dot indicates the type of coverage.

In 2022, the mean prevalence ratio was less than 1 in all regions, ranging from 0.68 in the MENA region to 0.95 in the LAC region (Table 1), indicating that pregnant women typically had lower HIV prevalence than women 15–49 yrs. The ESA and WCA regions had mean prevalence ratios of 0.72 and 0.83, respectively (Table 1). WCA had a higher proportion of outliers than ESA (Fig. 2; ESA: 6/46; WCA: 5/25). The regional mean ART coverage prepregnancy ratio was lowest in WCA at 0.40, and less than 1 for all regions, except WCENA and EECA where it was 1.22 and 1.06, respectively (Table 1). Twenty-two countries had ART coverage prepregnancy ratios above 1 (Fig. 3). The mean ART coverage prepregnancy ratio across all locations was 0.83 and the mean PMTCT coverage ratio was 1.21, and in all regions, the mean PMTCT coverage ratio exceeded the ART coverage prepregnancy ratio. For all regions except WCA (0.85), the PMTCT coverage ratio exceeded 1.

**TABLE 1.** Typical Ranges of Prevalence Ratios (Defined as HIV Prevalence Among Pregnant Women to HIV Prevalence Among Women 15–49 yrs), ART Pre-pregnancy (Defined as ART Coverage Among PWLHIV at First ANC to ART Coverage Among WLIHV 15–49 yrs), and PMTCT (Defined as ART Coverage Started Before or During the Pregnancy Among PWLHIV to ART Coverage Among WLHIV 15–49 yrs) Ratios

Ratio	UNAIDS Region	Mean (SD)	Typical Range (0.75–×1.25 mean)	Typical Local Adjustment Factor*
Prevalence	Eastern and southern Africa	0.72 (0.20)	0.54–0.90	1.02
	Western and central Africa	0.83 (0.24)	0.62–1.04	1.12
	Latin America and Caribbean	0.95 (0.29)	0.71–1.18	1.62†
	Asia and Pacific	0.73 (0.41)	0.55–0.92	1.07†
	Eastern Europe and central Asia	0.73 (0.24)	0.55–0.91	1.28†
	Western and central Europe and North America	0.70 (0.17)	0.53–0.88	1.07†
	Middle East and northern Africa	0.68 (0.21)	0.51–0.84	1.08†
ART prepregnancy coverage	Eastern and southern Africa	0.69 (0.17)	0.52–0.87	0.99
	Western and central Africa	0.40 (0.16)	0.30–0.50	1.27
	Latin America and Caribbean	0.67 (0.32)	0.50–0.84	1.69†
	Asia and Pacific	0.81 (0.36)	0.61–1.01	1.21†
	Eastern Europe and central Asia	1.06 (0.25)	0.79–1.32	1.29†
	Western and central Europe and North America	1.22 (0.42)	0.91–1.52	1.08†
	Middle East and northern Africa	0.84 (0.45)	0.63–1.05	1.37†
PMTCT coverage	Eastern and southern Africa	1.04 (0.26)	0.78–1.30	1.02
	Western and central Africa	0.85 (0.32)	0.64–1.06	1.19
	Latin America and Caribbean	1.19 (0.48)	0.89–1.48	2.14†
	Asia and Pacific	1.08 (0.55)	0.81–1.35	1.05†
	Eastern Europe and central Asia	1.51 (0.40)	1.13–1.89	1.61†
	Western and central Europe and North America	1.47 (0.49)	1.10–1.84	1.16†
	Middle East and northern Africa	1.34 (0.95)	1.00–1.67	1.20†

* The typical local adjustment factor is the weighted mean local adjustment factor for all locations in a region, with the weights representing how close the location's prevalence, ART pre-pregnancy coverage, or PMTCT coverage ratio is to the region's average. The method for calculating detailed in section S1 and Table S4 of Supplemental Digital Content, http://links.lww.com/QAI/C344.

† Not calibrated to routine HIV testing done at ANC data.

### Atypical Ratios Within Regions

All regions had countries with prevalence ratios outside of the regional typical range (Table 1). Globally, 19 of 154 locations (11 national locations, 8 subnational locations across Ethiopia, Kenya, and Zimbabwe) reported more PWLHIV receiving ART than the estimated number of PWLHIV in 2022. Of these 19 locations, 2 countries reported more pregnant women on ART before the current pregnancy than total estimated PWLHIV. Every region had countries with ART pre-pregnancy ratios outside of 0.75–1.25 times the mean ART pre-pregnancy ratio regional range (Fig. 2).

### Burkina Faso Case Study

We applied the data review steps described in Figure 1 to the Burkina Faso Spectrum-AIM file. Burkina Faso calibrated HIV prevalence among pregnant women to HIV prevalence from ANC-RT data. Prevalence from routine ANC testing in 2022 was 1.19%; Spectrum estimated HIV prevalence among all women 15–49 yrs was 0.7% (0.6%–0.9%). This differed from the typical relationship where prevalence among pregnant women was less than HIV prevalence among all women.^14^ Step 1 of the process (Fig. 1) revealed that the prevalence ratio in Burkina Faso (1.56) exceeded the typical range for WCA (0.62–1.04, Fig. 2). Step 2 identified Burkina Faso LAF parameter of 2.15 was greater than the typical LAF in the region (1.12, Table 1). The third step situated Burkina Faso among countries with a national household survey that informed FRR parameter estimates in Spectrum-AIM, indicating that the default FRR parameters should represent the relative fertility of WLHIV in Burkina Faso. Because both the prevalence ratio and LAF were higher than typical for WCA, we determined that the ANC-RT reported HIV prevalence was higher than expected for a country in WCA.

**FIGURE 4.**
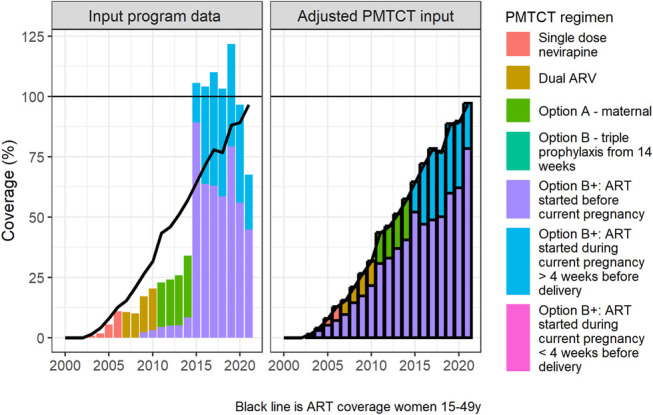
Adjustment of PMTCT program data for Burkina Faso, shown as the implied percentage coverage relative to Spectrum-AIM estimated PWLHIV. Bars show estimated PMTCT coverage by regimen; the line shows ART coverage among all WLHIV 15–49 yrs for comparison. Left: using original PMTCT program data; right: adjustment to address outcomes of the alignment assessment (Fig. 1), as described in section S2 of Supplemental Digital Content, http://links.lww.com/QAI/C344, bold outlining indicates a modeled value.

The Burkina Faso ratios for ART coverage prepregnancy (0.47) and PMTCT coverage (1.00) were both within typical ranges for WCA (0.30–0.50 for ART prepregnancy and 0.64–1.06 for PMTCT ratios in WCA, Table 1). We did not find inconsistencies in ART coverage among WLHIV 15–49 yrs in step 4. The steps to detect inconsistencies in PMTCT program data inputs (5 and 6) indicated that the number of WLHIV on PMTCT exceeded the modeled number of PWLHIV in each year between 2015 and 2020 (Fig. 4). The discrepancy between pregnant women reported on ART at delivery and estimated number PWLHIV was largest in 2019, when the reported number of PWLHIV on ART at delivery treatment was 1.67 times Spectrum-AIM's estimate of PWLHIV. Between 2014 and 2015, the reported number of WLHIV receiving PMTCT tripled from 4285 to 12,937.

The steps outlined in Figure 1 suggested 2 potential adjustments to reconcile Burkina Faso estimates of HIV prevalence among pregnant women: (1) HIV prevalence among pregnant women compared with all women 15–49 yrs was higher than typical for countries in WCA and (2) the number of PWLHIV receiving treatment was inconsistent with the estimated PWLHIV. Both discrepancies could be explained by the inaccurately large number of reported PWLHIV on ART before current pregnancy, which could indicate an issue with the ANC data quality and could occur if some PWLHIV on ART were double counted. Per the guidance in steps 1, 2, and 3, we decreased Burkina Faso LAF from 2.15 (fit to ANC-RT data) to 1.12 (WCA regional estimate), as an initial step to align Burkina Faso prevalence ratio with regional estimates. This choice assumes that estimates of HIV prevalence among women 15–49 yrs, informed by statistically representative national household surveys, are more accurate than the ANC HIV prevalence measured from routine antenatal care reporting data. During step 4, we did not change ART coverage among women 15–49 yrs, because we did not identify inconsistencies in ART program data for women 15–49 yrs. Because the PMTCT ratio was within the range of typical values for WCA (Table 1), we applied Burkina Faso program distribution of women on ART before pregnancy versus during pregnancy to Burkina Faso ART coverage among women 15–49 yrs to produce revised number of women receiving PMTCT and PMTCT coverage for Burkina Faso (Figure 4, and see S2 and Fig. S6, Supplemental Digital Content, http://links.lww.com/QAI/C344). Combined, reducing the LAF and updating the number of women receiving antiretrovirals for PMTCT resulted in a new prevalence ratio of 1.07, consistent with other countries in the region.

## DISCUSSION

Our study compared ratios of HIV prevalence and treatment coverage among pregnant women with all adult women within global regions using an algorithmic approach and the 2023 Spectrum-AIM files. We developed an algorithm to guide a data review process for Spectrum-AIM users to assess the relationship between ANC and ART related program data inputs and assumptions about fertility among WLHIV. This algorithm relies on 3 ratios (prevalence, ART coverage prepregnancy, and PMTCT coverage), prompting users to interrogate discrepancies between program and surveillance data and Spectrum-AIM assumptions and to align estimates of HIV prevalence among pregnant women and ART coverage at delivery with other locations in the region.

HIV prevalence among pregnant women in 2022 was lower than among all women 15–49 yrs in all regions, typically by 20%–30%. Prevalence ratios varied less across countries in regions with larger HIV epidemics compared with regions with transmission more concentrated among key populations and their partners. The greater variation in the latter is likely due to HIV transmission and primarily occurring in selected populations, which may have different fertility patterns than the general population, variations in surveillance data generalizability (eg, more risk-based HIV testing at ANC), and difference in estimation approaches (see Table S1, Supplemental Digital Content, http://links.lww.com/QAI/C344). Regarding the difference in surveillance data reliability, coverage of HIV testing at ANC was lower in the 24 countries outside of SSA, which fit the LAF to ANC-RT data (median coverage 71%), than in SSA countries (median coverage 78%), indicating ANC-RT HIV prevalence may not be as representative of all pregnant women in countries outside Africa.

In ESA, WCA, LAC, AP, and MENA, ART coverage before pregnancy was lower than ART coverage among all adult WLHIV, but by varying degrees, ranging from 0.4 in WCA to 0.84 in MENA (Table 1). In contrast, ART coverage before pregnancy was higher than ART coverage among all WLHIV 15–49 yrs in EECA (1.06) and WCENA (1.22) (Table 1). All countries in EECA and WCENA with ART prepregnancy ratios greater than 1 were estimated with the CSAVR model, which uses AIDS-related deaths reported through vital registration to estimate age-specific HIV prevalence (see Table S1, Supplemental Digital Content, http://links.lww.com/QAI/C344).^6^ Assessing typical results for the prevalence, ART coverage prepregnancy, and PMTCT ratios for countries without data that directly measures HIV prevalence among pregnant women requires more nuance than for countries with ANC-RT data.

Interpreting outlier ratios involves scrutinizing both local HIV surveillance data for total population prevalence estimation and its implications for estimating HIV prevalence among pregnant women.

1. Countries should assess the reliability of data used to estimate age-specific HIV prevalence among adults, considering documented biases in HIV surveillance data.^29–34^ These biases included duplication in antenatal care clinic data because of patient mobility during pregnancy and where death registration data are used, the frequency of misclassification of cause of death when used.^34^
2. If data on WLHIV at ANC and receiving PMTCT are reliable and complete, yet ratios are outlying from regional patterns, we propose countries manually adjust the LAF to align the number of PWLHIV with the program reported number of PWLHIV on ART. The LAF needed to scale the estimated PWLHIV to program reported number of PWLHIV receiving ART may yield insight into the relative fertility of WLHIV in that setting.

This second step improves the consistency in national HIV estimation and helps Spectrum model developers and UNAIDS to customize FRRs for countries outside of SSA. Both national estimations and Spectrum-AIM regional default fertility would be improved if some or all countries conducted representative sampling of HIV prevalence during antenatal care.

Although the proposed ratios describe relationships between surveillance data, programmatic input, and model assumptions, they are intrinsically linked through the model and cannot be evaluated independently. Aligning 1 ratio with the mean regional value may cause another ratio to diverge from the regional value; balancing these ratios requires an iterative process of assessing the accuracy of different input data sources and making justified adjustments. Although there is no gold standard source to compare these ratios with, using ratios calculated from external sources (such as national PHIA and/or DHS household surveys) in conjunction with regional ranges to identify outliers is a starting point to scrutinizing implausible model inputs.

The Burkina Faso case study illustrates this. Burkina Faso prevalence ratio was higher than many locations in the WCA region and accompanied by a high LAF fit to national ANC-RT data. The atypically high prevalence ratio suggested that Burkina Faso ANC-RT data overstated HIV prevalence among pregnant women relative to all women 15–49 yrs. Previous studies found ANC testing clinics in Burkina Faso disproportionately serve urban areas with higher HIV prevalence among pregnant women than national HIV prevalence among pregnant women.^35^ The difference in clientele is corroborated by lower access to multiple ANC visits before delivery in rural areas of Burkina Faso.^35,36^ Ultimately, the Burkina Faso estimation team determined that the program data were not robust for use in HIV estimation, indicated by low concordance between prevalence values from routine program data and sentinel surveillance data.

We suggest Spectrum-AIM users complete this review during the annual UNAIDS-supported HIV estimation process. Countries in regions where routine HIV testing at antenatal care is universal should consider adjustment based on regional trends as illustrated for Burkina Faso. Spectrum-AIM users in countries with epidemics primarily among key populations and more heterogeneous fertility among WLHIV and access to care should prioritize insights about the local epidemic context and ANC coverage data to refine estimates of HIV prevalence and ART coverage among pregnant women.^37–40^ Where information on the fertility of key populations exists, it may be useful to assess whether region, epidemic type, or HIV status is the largest determinant of fertility patterns among WLHIV. For all countries, we recommend triangulating results with additional valid data sources, including testing of HIV exposed infants with mother infant pairing and the number of children living with HIV receiving ART.^41–43^ Investing in validation with external data not input to the Spectrum-AIM model will produce more accurate estimates of HIV prevalence among pregnant women and number of vertical HIV transmissions. The work presented here is complemented by triangulation at the national scale to improve PMTCT programs.^44–48^ Such data triangulation is well illustrated by Sibanda et al,^44^ who integrated data from 4 sources (including Spectrum-AIM results) to identify gaps in the PMTCT care cascade in Zimbabwe, improving program results.

Our work provides recommendations and an algorithm to examining and, where needed, improving estimates of HIV prevalence among pregnant women within the Spectrum-AIM modeling process. We provide a path forward for targeted data collection and quality review for further refining these parameterizations. Future work could expand this analysis to include metrics measured through other data systems, such as early infant diagnosis, child ART registries, and vital registration. Improved estimates of the number of pregnant women with HIV will aid measuring and addressing PMTCT needs, thereby decreasing instances of vertical transmission, toward elimination of maternal to child transmission.

## Supplementary Material

SUPPLEMENTARY MATERIAL
